# Extracellular Matrix Remodelling in the Human Sural Nerve in Peripheral Vascular Disease

**DOI:** 10.3390/medicina62040737

**Published:** 2026-04-12

**Authors:** Braca Kundalić, Vladimir Petrović, Aleksandra Antović, Ivana Graovac, Slađana Ugrenović

**Affiliations:** 1 Department of Anatomy, Faculty of Medicine, University of Niš, 18000 Niš, Serbia; ivana.graovac@medfak.ni.ac.rs (I.G.); sladjana.ugrenovic@medfak.ni.ac.rs (S.U.); 2 Department of Histology and Embryology, Faculty of Medicine, University of Niš, 18000 Niš, Serbia; vladimir.petrovic@medfak.ni.ac.rs; 3 Department of Forensic Medicine, Faculty of Medicine, University of Niš, 18000 Niš, Serbia; aleksandra.antovic@medfak.ni.ac.rs

**Keywords:** extracellular matrix, peripheral nerve, peripheral vascular disease, collagen I, collagen IV, laminin, morphometry, human sural nerve, perineurium, endoneurium

## Abstract

*Background and Objectives*: Peripheral nerve adaptation to different pathological conditions is accompanied by the remodelling of the nerve’s extracellular matrix (ECM). Ischemic conditions caused by peripheral vascular disease are known to affect the function of peripheral nerves; however, the morphological changes to their ECM remain insufficiently examined and understood. Bearing in mind that alterations in collagen I, collagen IV, and laminin content may compromise peri- and endoneurial integrity, the aim of our study was to analyse whether peripheral vascular disease (PVD) induces distinct ECM alterations in the human sural nerve compared with the adaptive remodelling observed in ageing. *Materials and Methods*: The study aimed to determine the amount of type I and IV collagen and laminin in the perineurium and endoneurium of human peripheral nerves from patients with PVD and to compare the results with those of the age-matched controls. Twenty human sural nerves were harvested from cadavers and amputated limbs—10 from each—and were further distributed into two age groups: below and over 75 years of age. The sural nerve tissue samples were stained immunohistochemically for collagen I, collagen IV, and laminin. We measured the percentage content of these ECM components in the perineurium and endoneurium. For morphometric analysis, we used ImageJ software v1.54d. *Results*: Perineurial collagen type I and laminin were decreased in the older PVD group, relative to both the younger PVD and the older age group. Within the endoneurium, the expression of collagen type IV was higher in older PVD patients, while both collagen type I and laminin were deposited in lower amounts in the same group compared with the younger PVD group. *Conclusions*: These findings suggest that age-related ECM remodelling in the peripheral nerve may be impaired under ischemic conditions in older adults, with implications for surgical grafting strategies or neural conduit therapies aimed at promoting functional regeneration.

## 1. Introduction

The extracellular matrix (ECM) of the peripheral nerve is a dynamic and complex network of proteins and polysaccharides that provides not only structural support but also actively regulates cellular functions, including adhesion, migration, proliferation, differentiation, and cell survival. Collagen I, collagen IV, fibronectin, and laminin are considered the four major components of the ECM in peripheral nerves [1]. Collagen I fibrils are found abundantly in the epineurium, between multiple concentric perineurial layers, and in the surrounding axons [2]. Collagen IV and laminin have been observed in the basal lamina of perineurial and Schwann cells, as well as around blood vessels [3,4]. Under the influence of the ischemic damage that is characteristic of peripheral vascular diseases, significant changes in the composition, organisation, and function of the ECM take place, resulting in direct consequences for the functioning of Schwann cells, myelination, and the ability to regenerate peripheral nerves [1,5,6,7]. After ischemic injury, the ECM is remodelled, creating a microenvironment that can be either supportive or inhibitory for axonal regeneration and remyelination [1,5].

The vascular network of the peripheral nerve is delicate, and structural alterations to the nerve may affect blood supply [8]. A clinical condition that affects blood vessels in the limbs and commonly leads to ischemia-induced peripheral neuropathy is peripheral vascular disease (PVD) [9]. The most common ischemia-induced histopathological changes in peripheral nerves caused by vasculitic or vascular neuropathy include fibrous necrosis with inflammatory infiltrates, sub-endoneurial oedema, perivascular microfasciculation [10], and axonal loss [11]. Biopsy of the sural nerve is an important differential diagnostic procedure used to establish a proper diagnosis of vascular neuropathy, a condition predominantly characterised by vasculitis and injury to blood vessels [10].

Regarding surgical approaches for injured peripheral nerves, the sural nerve graft is considered an optimal donor for autologous grafting and the gold standard in peripheral nerve reconstruction [10,12,13]. It enables regeneration of the target nerve by acting as a scaffold and providing the Schwann cells needed for repair [10]. Promising second-line therapeutic options include artificial nerve conduits, which are scaffolds enriched with stem cells [14] and ECM proteins, such as collagen and laminin [8,15]. Vascularised nerve grafts have been suggested to address the challenges posed by poorly vascularised recipient tissues [16]. Using this type of grafting, the sural nerve is usually harvested as a free vascularised nerve graft and transferred together with the superficial sural artery or as an arterialised graft with the lesser saphenous vein [16]. Nevertheless, if the nerve vasculature has already been compromised, a lack of nutrients may lead to axonal degeneration [17], the outcome of which depends on the duration of ischemia. Nerves can tolerate ischemia for 1–3 h [18], but long-term injury may compromise the reversibility of morphological changes. This makes ischemia models difficult to set up, even more so in the case of models of chronic ischemia [18].

In a previous morphometric study of PVD-affected sural nerves, we found a thickening of the perineurial sheath and reported no significant differences in the content of total endoneurial connective tissue between the PVD and control groups during ageing [19]. Following these results, we suggested further studies on the ECM components involved in its remodelling. To address these challenges, this study aimed to quantitatively analyse the content of collagen I, collagen IV, and laminin in the ECM of the perineurium and endoneurium of the human sural nerve in patients with an established diagnosis of PVD during ageing.

## 2. Materials and Methods

### 2.1. Materials

The study included 20 sural nerves harvested during autopsies (10 samples) at the Department of Forensic Medicine, Niš, Serbia, and after below- and above-knee amputations (10 samples) at the Vascular Surgery Clinic, Clinical Centre Niš, Serbia. For the PVD group, the inclusion criterion was a confirmed diagnosis of peripheral vascular disease. The exclusion criteria for the PVD patients were neuropathies caused by other pathological conditions, such as diabetes mellitus or neurotoxic chemotherapy. The inclusion criteria for the cadaveric control group were an absence of previously diagnosed systemic, peripheral vascular, or nervous system disorders. The sural nerve samples were dissected in the area between the lateral malleolus of the fibula and the calcaneal tendon. A 5 cm long skin cut was performed, and the outer skin and subcutaneous layers were removed to approach the nerve next to the lesser saphenous vein. A part of the nerve trunk, 3 cm long, was harvested and fixed for 24–48 h in 10% neutral-buffered formalin within one hour after removal. All samples were divided into two age groups: younger (51–74 years old) and older (75–88 years old). The average ages of the PVD patients were 60.8 ± 7.4 years (ranging from 52 to 74) and 80.6 ± 4.7 years (ranging from 75 to 88), respectively. The average ages in the control group were 62.6 ± 7.5 (ranging 51–73 years) and 80.4 ± 3.8 years (ranging 75–86 years).

### 2.2. Immunohistochemistry

After fixation, the tissue sections were subsequently dehydrated in an ascending alcohol series (75%, 96%, and 100%). The nerve samples were cleared in xylene and embedded in paraffin. The tissue sections were cut on a Leica microtome at 5 µm thickness and mounted on slides. After deparaffination in the thermostat and xylol, the tissue samples were rehydrated in distilled water. Antigen retrieval was performed with incubation with trypsin at 42 °C for 90 min. The tissue sections were subsequently quenched with 3% hydrogen peroxide for 10 min at room temperature to remove the endogenous peroxidase. The tissue slides were incubated overnight with the primary antibody at 4 °C. The primary antibodies used in the study, their supplier, and their respective dilutions are listed in Table 1. The secondary antibody, followed by streptavidin–HRP (horseradish peroxidase), was applied to the tissue sections, and each was incubated at room temperature for 30 min. Between steps, the tissue was rinsed with Tris buffer. To visualise the immunohistochemical reaction, we used diaminobenzidine as a chromogen. The tissue slides were then counterstained with hematoxylin, subsequently dehydrated in an ascending series of alcohols, cleared in xylene, and mounted using Canada balsam.

### 2.3. Morphometric Analysis

Images of the histological slides were taken using an Olympus BX50 light microscope (Olympus, Tokyo, Japan) equipped with a Leica DFC295 digital camera (Leica Microsystems, Wetzlar, Germany) at the Department of Histology and Embryology, Faculty of Medicine, University of Niš. ImageJ software v1.54d was used for morphometric analysis. Ten fields of interest per slide (fifty per group), each in the perineurium and the endoneurium, were randomly selected and taken with a digital camera under ×800 magnification. The 24-bit RGB (Red, Green, and Blue) images were processed and analysed by ImageJ software (https://imagej.net/ij/index.html (accessed on 23 April 2023)). After setting global calibration in ImageJ using the option Analyze/Set Scale, each original image was duplicated before the analysis using the option Image/Duplicate. Sampling of perineurial content only was preceded by selecting the area of interest using the “Polygon selections” option, after which the image background was removed using the Edit/Clear Outside option. The area of the selected region of interest was measured using the option Analyze/Measure. Further steps were the same for the sampling of endoneurial content. The Image/Adjust/Color Threshold plug-in was activated, with the Black and White (B&W) option selected as the Threshold colour. Using the Polygon selection from the menu, at least five positive pixels (brown) were selected from the duplicated image. Using the option Sample in the Threshold Color window, only positive pixels were retained in the image (black pixels). The obtained image was compared with the original, and if the processing was satisfactory, the duplicate images were converted to binary using the Process/Binary/Make Binary option. In the programme menu, the Area Fraction option was checked under Analyze/Set measurements. After conversion of the analysed image into a binary one using the option Analyze/Measure, we obtained the result that represented the percentage (%) of positive pixels within the analysed area (image). Thereby, we acquired the percentage (%) values of collagen I, collagen IV, and laminin in each examined field. The collagen type I, type IV, and laminin percentage contents of the peri- and endoneurium for a single case were obtained as the mean of 10 analysed fields of vision [20].

### 2.4. Sample Size Determination and Statistical Analysis

Regarding effect sizes, we perform a pilot study on three nerves per group, a priori. Ten fields of vision per nerve sample were examined for the collagen IV in endoneurium (30 in total per group). After calculating the mean values and standard deviations for each group, the data were entered into G*Power 3.1.9.7. Since the study design was such that we opted to determine possible differences between all groups at once, we chose the F tests as a Test family, ANOVA: Fixed effect, omnibus, one-way for the statistical test. Alpha err prob (Alpha error probability) was set to 0.05, power was set to 0.8, and the number of groups was 4. After entering the mean values for each group and the pooled SD, we obtained an effect size (f) of 1.14, indicating that the total number of samples required per group to ensure statistical validity is 16 (4 per group). To strengthen the data and in line with other morphological analyses, the authors decided to examine 5 samples per group (20 in total).

Data were processed using the IBM SPSS statistical package, version 26. Descriptive statistics included a normality assessment and calculation of the mean and standard deviation. An assumption of homogeneity of variances was tested and found to be violated using Levene’s test. Differences between the groups were therefore evaluated by Welch’s ANOVA, followed by the Games–Howell post hoc test, a robust test of choice for heteroscedastic data and smaller sample sizes. All differences were deemed statistically significant at values of *p* < 0.05.

## 3. Results

The results of our study indicate that the peri- and endoneurial extracellular matrices of human sural nerves affected by peripheral vascular disease show significant alterations compared with age-matched healthy controls. We have already described the morphological changes in the human sural nerves of PVD patients in our previous paper [19]. All numerical data, with detected statistically significant differences, are presented in Table 2 and Table 3. The values of the content of examined ECM components are presented as percentages in the form of mean values with standard deviations and 95% confidence intervals.

### 3.1. Perineurium

**Figure 1:**
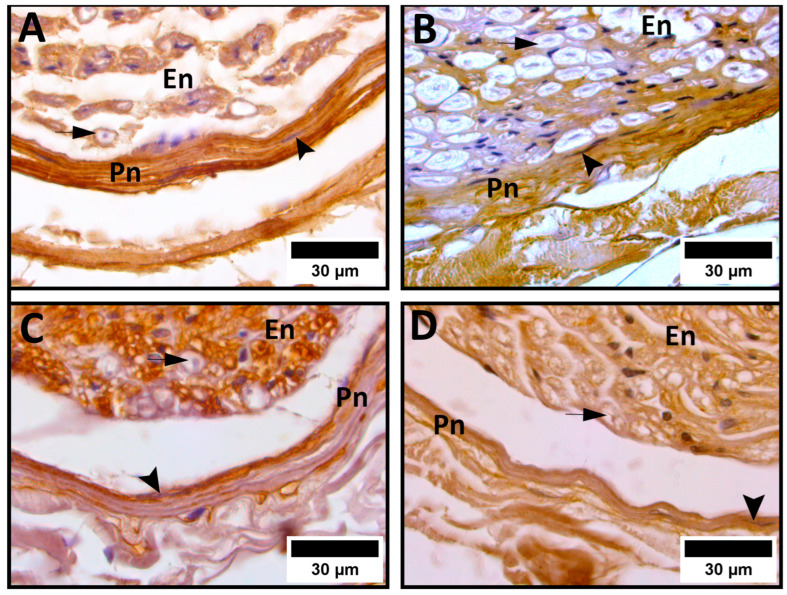
Immunohistochemical expression of collagen I (**A**,**B**) and laminin (**C**,**D**) in the perineurium. (**A**) Cross-section of the sural nerve of a 78-year-old peripheral vascular disease (PVD) patient; (**B**) cross-section of the sural nerve from an 81-year-old cadaver; (**C**) cross-section of the sural nerve of a 52-year-old PVD patient; and (**D**) cross-section of the sural nerve from a 58-year-old cadaver. Significantly higher expressions of collagen I and laminin are noted in PVD patients (**A**,**C**) vs. cadaveric controls (**B**,**D**). En—endoneurium; Pn—perineurium; arrowhead—perineurial cell; arrow—myelinated axon.

The results of our analysis of the percentage content of ECM components in the perineurium are given in Table 2. The collagen I content was higher in the PVD group compared with the age-matched controls. Within the PVD group, a statistically significant decrease in collagen I was evident in older PVD patients compared with younger patients. Compared with the older control group, older PVD patients had a statistically significant increase in collagen I expression. Statistical significance was also observed between the younger PVD and control groups, with PVD patients exhibiting a significantly higher collagen I content (Figure 1A,B).

It is intriguing that the overall content of perineurial collagen IV showed consistent values across the examined groups. No significant differences were detected between any pairs when the groups were compared statistically.

Higher values of the overall content of perineurial laminin were evident in the PVD groups. Within the PVD groups, we found a statistically significant decrease in laminin in older PVD patients compared with younger patients. Compared with the older control group, older PVD patients exhibited a statistically significant increase in laminin expression. A similar pattern was observed between the younger PVD and control groups, with laminin content significantly higher in PVD patients (Figure 1C,D).

### 3.2. Endoneurium

**Figure 2:**
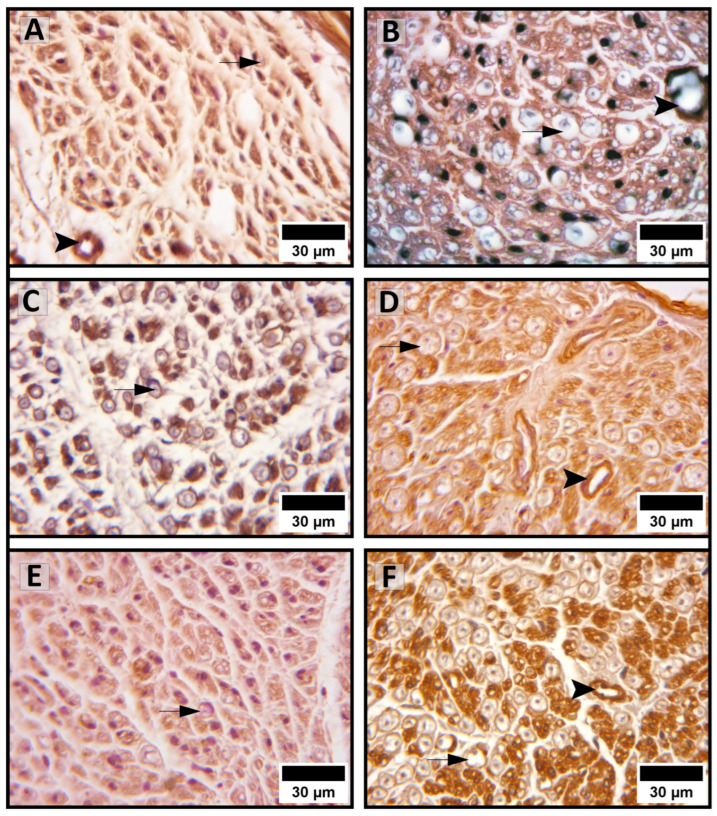
Immunohistochemical expression of collagen I (**A**,**B**), collagen IV (**C**,**D**), and laminin (**E**,**F**) in the endoneurium. (**A**) Cross-section of the sural nerve of an 84-year-old PVD patient; (**B**) cross-section of the sural nerve of a 74-year-old PVD patient; (**C**) cross-section of the sural nerve of a 78-year-old PVD patient; (**D**) cross-section of the sural nerve from a 75-year-old cadaver; (**E**) cross-section of the sural nerve of a 75-year-old PVD patient; and (**F**) cross-section of the sural nerve from a 75-year-old cadaver. Significantly lower reactivities of collagen I, collagen IV, and laminin are expressed in older PVD patients (**A**,**C**,**E**) vs. younger PVD patients (**B**) and age-matched controls (**D**,**F**), respectively. Arrowhead—endoneurial blood vessel; arrow—myelinated axon.

The values of endoneurial content of ECM components are presented in Table 3. The pattern of endoneurial collagen I expression was similar across the same age groups in PVD and healthy controls. We found a statistically significant decrease in collagen I content in older PVD patients compared with younger patients (Figure 2A,B). There was no statistical difference between the older PVD and older control groups, or between the younger PVD and control groups.

The overall content of endoneurial collagen IV was lower in the same age groups in PVD than in the healthy controls. Within the PVD group, our results indicate a significant increase in collagen IV in older PVD patients compared with the younger ones (Figure 2C,D). Also, the younger and older control groups had significantly higher collagen IV content compared with that in the older and younger PVD groups, respectively.

The content of endoneurial laminin was overall lower in the same age groups in PVD than in the healthy controls, similarly to collagen IV. However, within the PVD group, our results indicate a statistically significant decrease in laminin in older PVD patients (Figure 2E,F). Also, the values for laminin content were significantly lower in the older PVD group compared with the older control group.

## 4. Discussion

The results of our study showed alterations in peri- and endoneurial extracellular matrix components in the connective tissue sheaths of human peripheral nerves from patients with peripheral vascular disease, as compared with age-matched healthy controls. Perineurial collagen I and laminin content were significantly higher overall in the PVD group compared with the control group. However, within the PVD groups, older patients showed significant decreases in the production of these two molecules. Conversely, perineurial collagen IV values showed no statistically significant differences across the examined groups. Within the PVD groups, endoneurial collagen I and laminin decreased significantly in older PVD patients, while collagen IV was significantly higher in patients over 60 years of age.

Depending on the type of collagen, ageing and injury may affect its production differently. Collagen I is produced by fibroblasts and has a role in maintaining tissue function, while collagen IV is essential for the proper formation of basement membranes. The proper temporal distribution and ratio of collagen types I and IV in the ECM microenvironment are crucial for normal tissue function and regeneration. In the early post-injury phase, expression of collagen I is elevated in order to stabilise the ECM for axonal elongation and regrowth. Once deposited, its levels decrease in favour of remodelling proteins [21]. Collagen IV interacts with fibroblasts, which mediate ECM remodelling, so this type of collagen may be more critical for nerve tissue repair and wound healing; however, the excessive deposition by fibroblasts may promote scar formation and impair favourable outcomes [1].

Our results indicate greater collagen I deposition in the ischemic perineurium than in the control groups. However, its production in older PVD patients is significantly lower compared with that in their younger counterparts. A similarly significant ageing-induced decline in the collagen I content was also noted in the endoneurium of sural nerves in the PVD group. The perineurial fibroblasts exposed under continuous hypoxia for 48 h were shown to induce collagen I expression, compared with the control group with normoxia [22]. Persistent endoneurial hypoxia was attributed to nerve fibrosis, increased metabolic needs, and dysfunction of microvasculature [22]. Various data indicate a relationship between the extent of ischemia and the lesion degree. Ischemic model on the sciatic nerve showed deformed fibres with different thicknesses of myelin sheath but no changes in nerve fibre density after 30 days, which was in contrast to the morphological alterations in the tibial nerve, with myelin loss and axonal atrophy on the 15th day post-ischemia [23]. A clinical study on tibial nerves found significantly reduced fractional anisotropy values in patients with non-systemic vasculitic neuropathy compared with healthy controls. These reduced values were hypothesised to likely reflect axonal damage in this pathological state [24], as higher values are related to preserved myelin sheath, proper structural integrity, and the alignment of axons [25]. Cellular density or myelination actually affects the physical integrity of nerve tissue less than the composition of the ECM [26]. Schwann cell behaviour is tightly regulated by the characteristics of the ECM, particularly through changes in collagen composition. Collagen types I and IV shape the stiffness and elasticity of the microenvironment, which in turn influences Schwann cell adhesion, migration, and myelination [27]. During maturation, increased collagen deposition and remodelling of the basal lamina elevate ECM stiffness, supporting myelin production and axonal organisation and providing a favourable microenvironment for nerve signal transmission and lasting nerve homeostasis [5,28]. However, after nerve injury, excessive collagen accumulation and altered laminin structure can create a fibrotic environment that impairs Schwann cell proliferation and axonal regrowth and may drive Schwann cell senescence and limit their regenerative potential [29]. To counteract these adverse effects, biomimetic scaffolds enriched with bioactive laminin cues have been developed to restore a supportive ECM, thereby enhancing Schwann cell alignment, migration, and differentiation for improved nerve repair [30].

We found no statistically significant differences in perineurial collagen IV content across the examined groups. On the other hand, although the content of endoneurial collagen type IV was overall higher in the control group than in PVD patients, our results show a statistically significant increase in endoneurial collagen IV in older PVD patients compared with younger ones. The results for the control group are consistent with those of our previously published study [20]. Data from the literature suggest that ischemic injury may increase the vulnerability of the endoneurium, characterised by pathological alterations of myelinated axons [17,31].

The increase in collagen IV content in long-term ischemia may result from stimulated proliferation of endoneurial fibroblasts and Schwann cells. Deposition of collagen IV following injury activates the inflammatory process, which may prevent axon regeneration by physical blockage and induce the production of growth-inhibiting factors [32]. Studies report higher immunofluorescence expression of collagen IV and concomitant fibronectin in post-ischemic mouse, rat, and sheep tissues, as well as in human post-stroke brain tissue after 3 weeks of ischemia [33]. Additionally, collagen IV has been found in large quantities 14 days after injury within the endoneurium [34]. These findings may be related to our results indicating collagen IV accumulation in the aged endoneurium following chronic ischemia.

The literature on the collagen IV content of different tissues after damage is inconsistent. A study on rats reported a post-stroke decrease in collagen IV by Western blot analysis in an experimental model of ischemia, which may correlate with a decrease in cerebral blood vessels [35]. Data indicating the contrary were presented in an immunohistochemical study and were explained either by a potential increase in protein degradation, thereby making proteins more available for measurement, or by overcompensation to stabilise damaged vasculature [36]. It was also suggested that 48 h hypoxia may induce overexpression of collagen IV in nerves [22]. In the sera of patients with lower-limb arterial disease, the levels of degraded α1 chains of collagen IV were found to be both increased and associated with disease progression, but it remains unclear whether this may be due to the statin therapy and array of associated clinical entities in these patients [37]. Statins have already been associated with lower matrix metalloproteinase (MMP)-9 activity [38]. Unfortunately, we had no data about statin therapy in the PVD patients in our study, so we can only hypothesise that the production of collagen IV and its enzymatic degradation within perineurial ECM are in a lasting “tug-of-war” in peripheral vascular disease, which could explain the insignificant differences we found in the perineurium.

The laminin content in PVD patients in our study was significantly higher in the perineurium, but lower in the endoneurium, compared with the healthy ageing group. The PVD group showed a significant decrease in laminin in older patients. These results are similar to the study on patients diagnosed with lower-limb arterial disease, which presented decreased levels of laminin in the basement membrane of arteries, supposedly due to the stronger MMP activity [37]. Laminin plays a key role in the early development of the basement membrane compared with collagen IV [39]. These two proteins are concomitant after injury, so laminin was found to increase in the ischemic penumbra after stroke [40,41], but is less expressed in the ischemic core due to the decline in the number of post-ischemic blood vessels [42]. Laminin was noted as being significantly deposited at 6 weeks post-injury, along with collagen I; however, conversely, laminin also exhibits an overall increase at 12 weeks. Its expression was also positively correlated to axon density, which has been suggested as being associated with its role in the differentiation of Schwann cells towards a myelinating phenotype [21].

PVD is a chronic condition that leads to tissue ischemia and is characterised by vascular remodelling. It was reported that ischemia led to a significant downregulation of genes associated with the protective functions of ECM molecules, with this effect being more pronounced in aged animals [43]. Many studies have reported that vascular remodelling in PVD depends on MMP activity, including that of MMP-2, MMP-9, and MMP-14, which degrade the ECM. The imbalance of MMPs has been implicated in various vascular disorders [44]; in particular, MMP-2 and MMP-9 activity contribute to the degradation of ECM components of the vasculature (such as collagen and laminin) due to impaired blood flow or pressure adaptation [45]. The permanent occlusion of the middle cerebral artery has been shown to upregulate genes responsible for MMPs and downregulate those for tissue inhibitors of metalloproteinases (TIMPs) [43]. TIMPs are a family of four proteins (TIMP-1/2/3/4) that regulate MMP activity and may be seen as a potential therapeutic choice [38]. Increasing TIMP-1, an MMP-9 inhibitor, is associated with increases in the number and density of axons, improved myelination, and decreased scar formation [46]. Conversely, MMP-9 in molar excess, but not MMP-2, targets and cleaves TIMP proteins [47]. Matrix metalloproteinases have a dual nature as acute disruptors of structures that degrade the matrix and chronic promoters of remodelling, contributing to fibrosis [38]. Additionally, MMP-9 has been shown to promote neurogenesis in post-ischemic repair but can exert detrimental effects as well, causing nerve demyelination and perineural net disruption [48]. The fibrosis process may be triggered by either the overproduction of ECM components or insufficient degradation due to an imbalance between MMP and TIMP activities [48].

Understanding the composition of the extracellular matrix (ECM) is fundamental to the development of effective nerve repair strategies. While autologous nerve grafting remains the surgical gold standard, its application is constrained by donor-site morbidity, tissue availability, and neuroma formation [49,50,51]. To overcome these drawbacks, nerve guidance conduits have been introduced as biomaterial-based alternatives that support axonal regrowth [50]. These conduits are engineered using ECM-derived components, such as collagen and laminin, incorporated in varying proportions to mimic the native microenvironment and thereby support axonal regeneration. Collagen, which makes up nearly half of nerve proteins, is widely used due to its biocompatibility, though its weak mechanical properties require reinforcement with synthetic polymers [52]. Collagen I enhances Schwann cell migration, angiogenesis, and axonal regeneration, improving recovery. Conversely, collagen IV promotes fibroblast recruitment and inflammation, hindering regeneration [1]. Laminin is generally considered permissive for axonal growth, but comparative studies suggest fibronectin-coated collagen scaffolds may outperform laminin in peripheral nerve repair [51]. The use of pharmacologic agents is another promising strategy, one where studies on the ECM content may provide further insight in terms of finding uses for antifibrotic agents in targeting ECM proteins in a peripheral nerve [22].

The limitations of this study primarily pertain to the use of cadaveric nerves and the number of tissue samples. Cadaveric nerves were obtained within 24–48 h post mortem, in accordance with regulatory requirements governing autopsy procedures. Previous work on post-mortem morphological changes in human peripheral nerves has demonstrated early myelin loss within 24 h and myelin splitting by 48 h, although smaller axons remain structurally preserved [53]. Cadaveric nerves are frequently employed as controls, given the absence of routine, non-invasive methods for harvesting healthy human nerve tissue. Furthermore, the sample size was restricted to five nerves per group, a common practice in morphological studies due to limited tissue availability, ethical considerations, and the technical complexity of the procedures. These constraints should be acknowledged when interpreting the findings, as they may influence the generalisability of the results.

## 5. Conclusions

The findings of our study present alterations in the ECM architecture associated with ageing in patients with peripheral vascular disease. Considering the functional interdependence between the ECM and nerve tissue components, a comprehensive understanding of ECM remodelling in advanced PVD may be of use for understanding the regeneration process in ischemic conditions and the design of advanced conduits that would facilitate successful nerve regeneration. Future investigations are required to elucidate potential quantitative modifications in other ECM constituents, their dynamic interplay, and the implications of maintaining peripheral nerve homeostasis.

## Figures and Tables

**Table 1:** Primary antibodies used in the study.

Primary antibody	Supplier	Dilution
Collagen I (rabbit polyclonal anti-collagen I antibody)	Abcam (Cambridge, UK) ab34710	1:400
Collagen IV (rabbit polyclonal anti-collagen IV antibody)	Abcam (Cambridge, UK) ab6586	1:400
Laminin (rabbit polyclonal anti-laminin antibody)	Abcam (Cambridge, UK) ab11575	1:25

**Table 2:** Percentage content of collagen I, collagen IV, and laminin in perineurium.

Perineurial Content	Group	N	Mean	SD	Lower Bound 95% CI (%)	Upper Bound 95% CI (%)
Collagen I (%)	PVD-y	5	13.155 ^a,b^	3.002	12.302	14.008
	PVD-o	5	11.203 ^b,c^	3.075	10.329	12.077
	C-y	5	9.976 ^a^	2.156	9.363	10.589
	C-o	5	8.019 ^c^	1.409	7.619	8.420
Collagen IV (%)	PVD-y	5	28.075	5.998	26.370	29.779
	PVD-o	5	29.763	5.279	28.262	31.263
	C-y	5	28.185	4.797	26.821	29.548
	C-o	5	30.420	4.017	29.278	31.562
Laminin (%)	PVD-y	5	14.138 ^d,e^	4.743	12.790	15.487
	PVD-o	5	10.327 ^e,f^	2.694	9.561	11.092
	C-y	5	10.994 ^d^	3.216	10.080	11.908
	C-o	5	7.089 ^f^	1.868	6.558	7.620

PVD-y: younger PVD group; PVD-o: older PVD group; C-y: younger control group; C-o: older control group; CI: confidence interval. Letters in superscript mark statistically significant differences at *p* < 0.05.

**Table 3:** Percentage content of collagen I, collagen IV, and laminin in endoneurium.

Endoneurial Content	Group	N	Mean	SD	Lower Bound 95% CI (%)	Upper Bound 95% CI (%)
Collagen I (%)	PVD-y	5	12.254 ^g^	3.738	11.192	13.317
	PVD-o	5	6.395 ^g^	1.861	5.866	6.924
	C-y	5	12.353	5.432	10.810	13.897
	C-o	5	6.062	2.720	5.289	6.836
Collagen IV (%)	PVD-y	5	8.602 ^h,i^	3.174	7.699	9.504
	PVD-o	5	13.356 ^i,j^	2.649	12.603	14.109
	C-y	5	16.155 ^h^	3.951	15.033	17.278
	C-o	5	20.172 ^j^	4.124	19.000	21.344
Laminin (%)	PVD-y	5	11.598 ^k^	3.124	10.711	12.486
	PVD-o	5	5.857 ^k,l^	1.791	5.348	6.367
	C-y	5	13.319	4.291	12.100	14.539
	C-o	5	9.088 ^l^	2.578	8.355	9.820

PVD-y: younger PVD group; PVD-o: older PVD group; C-y: younger control group; C-o: older control group; CI: confidence interval. Letters in superscript mark statistically significant differences at *p* < 0.05.

## Data Availability

The data presented in this study are available upon reasonable request from the corresponding author.

## Abbreviations

The following abbreviations are used in this manuscript:

ECM	Extracellular matrix
PVD	Peripheral vascular disease
HRP	Horseradish peroxidase
RGB	Red, Green, and Blue
CI	Confidence interval
MMP	Matrix metalloproteinase
TIMP	Tissue inhibitor of metalloproteinase
